# Arginine Adjunctive Therapy in Active Tuberculosis

**DOI:** 10.1155/2015/205016

**Published:** 2015-02-03

**Authors:** Aliasghar Farazi, Omid Shafaat, Masoomeh Sofian, Manijeh Kahbazi

**Affiliations:** ^1^Department of Infectious Disease, School of Medicine, Arak University of Medical Sciences, Arak 38159 34798, Iran; ^2^Tuberculosis and Pediatric Infectious Diseases Research Center, Arak University of Medical Sciences, Arak 38159 34798, Iran; ^3^School of Medicine, Arak University of Medical Sciences, Arak 38159 34798, Iran

## Abstract

*Background*. Dietary supplementation has been used as a mechanism to augment the immune system. Adjunctive therapy with L-arginine has the potential to improve outcomes in active tuberculosis.* Methods*. In a randomized clinical trial 63 participants with smear-positive pulmonary tuberculosis in Markazi Province of Iran were given arginine or placebo for 4 weeks in addition to conventional chemotherapy. The final treatment success, sputum conversion, weight gain, and clinical symptoms after one and two months were considered as primary outcomes and secondary outcomes were ESR, CRP, and Hg. Data were collected and analyzed with SPSS software (ver. 18).* Results*. Arginine supplementation reduced constitutional symptoms (*P* = 0.032) in patients with smear-positive TB at the end of the first month of treatment. Arginine treated patients had significantly increased BMI at the end of the first and second months of treatment (*P* = 0.032 and *P* = 0.04) and a reduced CRP at the end of the first month of treatment (*P* = 0.03) versus placebo group.* Conclusion*. Arginine is useful as an adjunctive therapy in patients with active tuberculosis, in which the effects are more likely mediated by the increased production of nitric oxide and improved constitutional symptoms and weight gain. This trial is registered with Clinical Trials Registry of Iran: IRCT201211179855N2.

## 1. Introduction

Tuberculosis (TB) is an important global health problem. About one-third of the population is estimated to be infected with* M. tuberculosis*. People with latent TB infection (LTBI) do not have symptoms of TB and are not infectious, but they are at risk of developing active disease and becoming infectious. Studies show that 5–20% of those infected will develop active TB at some point in their lifetime, with the majority developing TB disease within 2–5 years of the initial infection. In 2012, an estimated 8.6 million people developed TB and 1.3 million died from the disease [1]. Arginine, first discovered over 100 years ago, is a basic amino acid naturally ingested in our diets at a rate of 3–5 g/d [2]. Arginine is particularly rich in certain food products, such as meats and nuts. Arginine can be synthesized endogenously but, in children and in people with certain conditions (e.g., infection, trauma), arginine synthesis may become compromised. It should be noted that groundnuts (peanuts) contain 1 g of arginine per 30 g of biomass and they are affordable and readily available worldwide [3, 4]. Arginine as a semiessential amino acid was found to be necessary for adequate wound healing [5–7]. These observations were summarized in the statement that arginine was a “conditionally essential amino acid” which should be supplemented at times of physical stress such as after surgery or trauma [8, 9].

Numerous reports based on animal models suggest that nitric oxide is important for host resistance during the acute phase of TB [10, 11]. The role of nitric oxide in humans is controversial, although recent findings suggeste its role in human TB [12, 13]. Inducible nitric oxide synthase catalyzes the synthesis of nitric oxide and citrulline from L-arginine in macrophages activated by cytokines, such as TNF-*α* and IFN-*γ*. Nitric oxide is highly unstable and decays to its stable end products nitrate and nitrite, which are eliminated in the urine in as much as malnutrition, and reduced food intakes are associated with TB [14]. In a randomized clinical trial of TB patients without HIV infection, supplement therapy by arginine resulted in higher sputum conversion rates, faster reduction of symptoms (e.g., cough), and increase in weight gain. However, these improvements were not seen in patients with HIV in the same trial or another trial [15, 16]. A placebo-controlled randomized trial in central province of Iran was conducted to ascertain whether adjuvant arginine supplementation can improve the clinical outcome of pulmonary TB.

## 2. Materials and Methods

### 2.1. Patients

We designed a clinical randomized placebo-controlled trial in new cases of smear-positive TB that were recruited with informed consent from December 2012 to May 2014 at the Direct Observed Treatment Short-Course (DOTS) Clinics in Markazi province. The inclusion criteria were age of ≥15 years and smear-positive pulmonary TB, as recommended by the World Health Organization (WHO) for DOTS. The exclusion criteria were hospitalization, pregnancy, clinical signs of any comorbidity (such as diabetes mellitus, malignancy, hepatic or renal failure, HIV^+^, etc.) according to physician's judgement and patient's medical documents, patients who received L-arginine supplement during the past month, and allergic reactions to L-arginine. Smear positivity was defined as two of three positive morning sputum samples or one of three positive with a chest radiograph and clinical symptoms suggestive of pulmonary TB. Treatment success according to WHO guidelines was defined as the sum of the cases that were cured and that completed treatment. Sputum smear was recorded at the beginning, one and two months after treatment. Body mass index was recorded at baseline, one and two months after beginning treatment. Blood samples for C-reactive protein (CRP was measured by latex agglutination tests), Erythrocyte Sedimentation Rate (ESR by Westergren method), and hemoglobin concentration (Hgb was measured by hematology analyzer Sysmex K1000) were obtained at baseline, one and two months after starting treatment. Using a standard form, patients were initially interviewed regarding duration of clinical symptoms, one and two months later concerning the presence or absence of symptoms (cough, constitutional symptoms, and body mass index). All laboratory tests were measured in one laboratory with the same personnel and calibrated equipment and all clinical signs and symptoms were measured by one person.

### 2.2. Treatment Regimes

All treatment was done on an outpatient basis, according to the WHO recommendations. The chemotherapy consisted of isoniazid, pyrazinamide, rifampicin, and ethambutol during the intensive phase of 2 months followed by isoniazid and ethambutol to 6 months.

At initiation of anti-TB therapy, patients with TB were assigned by randomization into supplementation with identical capsules of L-arginine (Karen Pharma & Food Supplement Co., Iran) which contains 1000 mg pure L-arginine hydrochloride or placebo which contains 1000 mg sugar twice daily administered orally for 30 days in accordance with previous studies [15, 16]. The final treatment success, sputum conversion, weight gain, and clinical symptoms after one and two months were considered as primary outcomes and secondary outcomes were ESR, CRP, and Hgb.

### 2.3. Design and Statistical Methods

**Figure 1:**
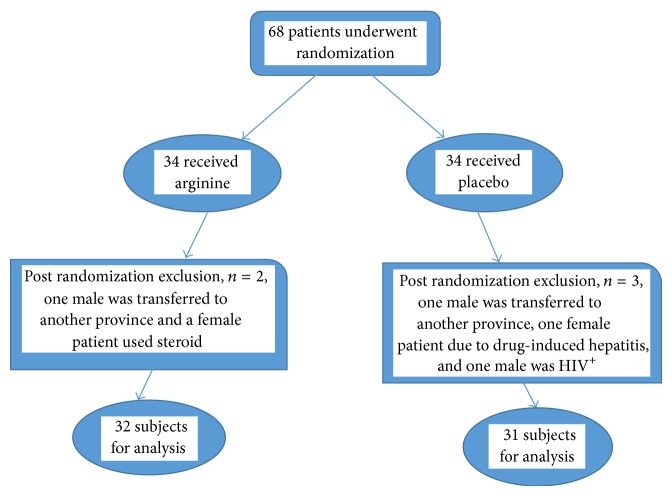
Study flow chart.

Sample size was calculated based on the published data of a resembling trial [17]. The formula of calculating sample size was

(1) $$\begin{array}{c} n=\frac{{\left(Z_{\alpha /2}+Z_{\beta }\right)}^{2}\times \left[p_{1}\left(1-p_{1}\right)+p_{2}\left(1-p_{2}\right)\right]}{{\left(p_{1}-p_{2}\right)}^{2}}, \end{array}$$

where *p*_1_ = %83.8 and *p*_2_ = %53.1 (*p*_1_ = cure rate in supplemented group and *p*_2_ = proportion of cure rate in placebo group). With a power of 85% and *α* level of 0.10, the sample size was calculated to be 29 for each group and taking into account 15% dropout, a total of 68 patients were enrolled in two groups of case and control. Patients were randomly assigned to either the arginine group or the placebo group. The randomization was 1 : 1 for the 2 groups and was performed by using random number tables, for the two assign regimens (Figure 1). Preparation of the randomization envelopes was performed by a member of the staff who was not directly involved in the study and a sealed copy of the treatment code was kept by the project leader until all data had been collected and analyzed.

Analyses were conducted using SPSS software (ver. 18) according to a prespecified plan. Outcomes were analyzed according to the arms to which the participant was originally assigned. Statistical analysis was performed by calculation of mean ± standard deviation; ANOVA (differences between group means), *t*-test and Mann-Whitney *U* test (differences between baseline and follow-up of all parameters in each group), and Fisher's exact test and chi-square test (comparison of categorical variables) were used; *P* values of <0.05 were significant.

## 3. Results

Of 68 eligible patients, 63 sputum AFB-positive patients were included in the study and 5 were excluded (two males were transferred to another province; one male was HIV^+^; one female patient due to drug-induced hepatitis; a female patient used steroid). Of the 63 patients that completed treatment, 32 patients were randomly assigned to the arginine group and 31 to the placebo group. In trial group 46.9% and control group 51.6% were male. The mean age of the trial group was 51.9 ± 22.8 and median was 52 years, in the control group mean age was 52.3 ± 21.7 and the median was 53 years. Sociodemographic characteristics of the study show that 41.3% of them belong to rural areas while 58.7% are from urban sector. There were no significant differences in any of the baseline characteristics between the arginine and placebo groups, including gender, age, body mass index (BMI), and laboratory characteristics (Hgb, ESR, and CRP) at the beginning of treatment. Baseline patient characteristics in two groups are shown in Table 1.

**Figure 2:**
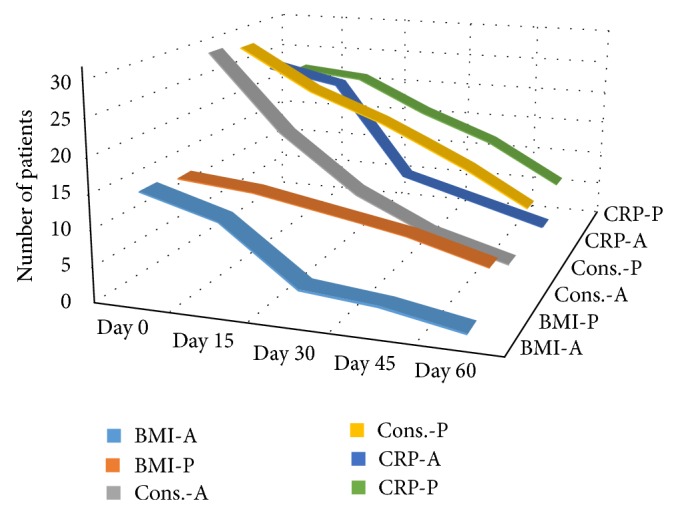
Trends in increased BMI, constitutional symptoms improvement, and decrease of CRP in patients of arginine group and placebo group.

Arginine supplementation reduced constitutional symptoms (*P* = 0.032) at the end of the first month of treatment. Arginine treated patients had significantly increased BMI at the end of the first and second months of treatment (*P* = 0.032 and *P* = 0.04) and a reduced CRP at the end of the first month of treatment (*P* = 0.03) versus placebo group. There was no meaningful difference in reducing of cough (*P* = 0.25 and *P* = 0.424), mycobacterial clearance in sputum smear (*P* = 0.254 and *P* = 0.15), decreasing rate of ESR (*P* = 0.561 and *P* = 0.258) and Hgb level (*P* = 0.08 and *P* = 0.064) between 2 groups at the end of the first and second months of treatment, reduced CRP (*P* = 0.118) at the end of the second months of treatment, and final treatment success (Table 2 and Figure 2).

## 4. Discussion

This study did not show any significant reduction of cough and mycobacterial clearance in sputum smear during the first 2 months of DOTS treatment in patients receiving arginine supplementation compared to placebo group and the final treatment success was similar in two groups but results of our study demonstrate that arginine was associated with some benefits such as reduced constitutional symptoms in patients with smear-positive TB at the end of the first month of treatment. Arginine treated patients had significantly increased BMI at the end of the first and second months of treatment and a reduced CRP at the end of the first month of treatment versus placebo group. The improved BMI, constitutional symptoms, and decreased CRP may be associated with increased arginine levels, which consequently have ergogenic potential because of their role in the secretion of endogenous growth hormone and their involvement in the synthesis of creatine, L-Arginine supplementation affects on CNS function, increasing nitric oxide and strengthen the immune system [18].

Nitric oxide acts as a cytotoxic agent in pathological processes and can therefore play a central role in the regulation of immune responses [19]. Nitric oxide bioavailability is impaired in pulmonary TB in proportion to clinical severity and is associated with delayed mycobacterial clearance [20, 21]. Hypoargininemia can develop when arginine catabolism exceeds endogenous synthesis and hypoargininemia has been demonstrated in TB [22]. In addition to immunological effects, L-arginine can influence antimycobacterial cell-mediated responses directly, being required for expression of the T cell receptor's CD3 zeta component [22]. Two trials have addressed L-arginine in TB, testing L-arginine hydrochloride (1 g) or arginine-rich food (peanuts). In the beginning, faster sputum-microscopy clearance and cough resolution were reported in the supplemented arm, but only among HIV^−^ participants [16]. The second study reported no significant benefits overall and found higher cure rates in HIV^+^ participants receiving the arginine-rich supplement [23]. Ralph et al. evaluated L-arginine with vitamin D as adjunctive TB therapies and at the doses evaluated, they could not demonstrate that these agents alone or in combination were associated with benefits in TB outcomes [24]. A previous study has shown that arginine supplementation led to decreased morbidity from infectious diseases in high-risk patients undergoing surgery [25]. The improved clinical outcome observed in TB patients was probably mediated by augmented production of NO induced by increased arginine intake. These results support a role for arginine supplementation aimed at enhancing the human antimycobacterial effect during the treatment of active TB. In our study no major side effects were reported in the arginine groups and L-arginine has few adverse reactions. Nausea and diarrhea have been reported infrequently. The important limitation in this study related to the lack of evaluation of concentration of arginine and nitric oxide, short length of observation, lack of sputum culture, and small sample size. We propose that further studies be conducted to investigate in larger samples with higher doses and longer duration of L-arginine.

## 5. Conclusion

Arginine is useful as an adjunctive therapy in patients with active tuberculosis, which the effects more likely to be mediated by the increased production of nitric oxide. This study showed that arginine is beneficial as supplement treatment in patients with active tuberculosis, an effect most likely improved constitutional symptoms, weight gain, and reduction of CRP despite the lack of effect on anemia, cough, sputum smear conversion, and final treatment success.

## Figures and Tables

**Table 1:** Clinical and laboratory findings in arginine and placebo group before treatment.

Sign and symptom	Group		*P* value
	Arginine *N* = 32 (%)	Placebo *N* = 31 (%)	
Age (mean ± SD)	51.9 ± 22.8	52.3 ± 21.7	0.943
Gender			
Male	15 (46.9)	16 (51.6)	0.704
Female	17 (53.1)	15 (48.4)	
Resident			
Rural	14 (43.7)	12 (38.7)	0.682
Urban	18 (56.3)	19 (61.3)	
Constitutional symptoms	30 (93.8)	29 (93.6)	0.976
Cough	29 (90.6)	28 (90.3)	0.968
BMI < 18.5	15 (46.9)	14 (45.2)	0.889
Tuberculin test >10 mm	27 (84.4)	26 (83.8)	0.96
Sputum smear >1+	13 (40.6)	13 (41.9)	0.912
Anemia^*^	17 (53.1)	16 (51.6)	0.904
↑ ESR^**^ mm/h	28 (87.5)	29 (93.6)	0.412
↑ CRP^***^	24 (75)	22 (71)	0.719

^*^Hg <13 g/dl in men and Hg <12 g/dl in women.

^**^The normal range (Westergren method) for males is 0–15 mm/h and for females is 0–20 mm/h.

^***^CRP >10 mg/L.

**Table 2:** Clinical and laboratory findings in arginine and placebo groups at the end of the first and second months^*^.

Sign and symptom	Group		*P* value
	Arginine *N* = 32 (%)	Placebo *N* = 31 (%)	
Constitutional symptoms			
First month	11 (34.4)	19 (61.3)	0.032
Second month	3 (9.4)	8 (25.8)	0.085
Cough			
First month	15 (46.9)	19 (61.3)	0.25
Second month	3 (9.4)	5 (16.1)	0.424
BMI < 18.5			
First month	4 (12.5)	11 (35.5)	0.032
Second month	1 (3.1)	6 (19.4)	0.04
Positive sputum smear			
First month	14 (43.8)	18 (58.1)	0.254
Second month	1 (3.1)	4 (12.9)	0.15
Anemia			
First month	6 (18.8)	12 (38.7)	0.08
Second month	2 (6.3)	7 (22.6)	0.064
↑ ESR mm/h			
First month	25 (78.1)	26 (83.9)	0.561
Second month	11 (34.4)	15 (48.4)	0.258
↑ CRP			
First month	8 (25)	16 (51.6)	0.03
Second month	2 (6.3)	6 (19.4)	0.118
Final treatment success (end of sixth month)	32 (100%)	31 (100%)	—

^*^Categorical variables were compared using the chi-square test or Fisher's exact test where appropriate. Continuous variables were compared using the *t*-test or the nonparametric Mann-Whitney *U* test according to data distribution.
